# Anticoagulant prescribing trends, bleeding events, and reversal agent use in pediatric patients: A retrospective, real-world study

**DOI:** 10.1371/journal.pone.0323137

**Published:** 2025-05-08

**Authors:** Sofia D’Abrantes, Christoph Male, Nick Brown, Mikael Bjursell, Philip Ambery, Anders Berggren, Ulrika Mattsson

**Affiliations:** 1 Data Sciences and Artificial Intelligence, R&D BioPharmaceuticals, AstraZeneca, Cambridge Biomedical Campus, Cambridge, United Kingdom; 2 Department of Pediatrics, Medical University of Vienna, Vienna, Austria; 3 Department of Women’s and Children’s Health, International Maternal and Child Health (IMCH), Uppsala University, Uppsala, Sweden; 4 Department of Paediatrics, Länssjukhuset Gävle-Sandviken, Gävle, Sweden; 5 Department of Child Health, Aga Khan University, Karachi, Pakistan; 6 Late-stage Development, Cardiovascular, Renal and Metabolism, R&D BioPharmaceuticals, AstraZeneca, Gothenburg, Sweden; Tokai University School of Medicine: Tokai Daigaku Igakubu Daigakuin Igaku Kenkyuka, JAPAN

## Abstract

This retrospective real-world study aimed to describe anticoagulant prescribing trends, particularly for factor Xa (FXa) inhibitors, bleeding events, and reversal agent use in pediatric patients to assess potential populations for clinical trials of the FXa inhibitor reversal agent andexanet alfa. Real-world health care data from the TriNetX Global Network and Optum’s deidentified Clinformatics^®^ Data Mart Database (CDM) were analyzed to identify patients aged <18 years old who were prescribed a direct oral FXa inhibitor, warfarin, or low-molecular-weight heparins from 2007 through 2024 (TriNetX, N = 59,780) or 2023 (CDM, N = 6470). The only anticoagulants prescribed to children were warfarin and/or low-molecular-weight heparins in 2007 and 2008 in TriNetX and from 2007 through 2010 in CDM. Prescriptions of the FXa inhibitor rivaroxaban increased from 0.4% (2009) to 18.0% (2023) in TriNetX and from 0.8% (2011) to 34.0% (2023) in CDM, with similar trends for apixaban. Relevant bleeding was reported in 9.4% of patients prescribed an FXa inhibitor in TriNetX; ≤ 0.1% of patients received andexanet alfa the day of a bleed. Among patients prescribed an FXa inhibitor, ≤ 0.1% in TriNetX and 0 in CDM received andexanet alfa the day of surgery. Direct oral FXa inhibitor use in children is growing, as is the potential for associated bleeds; however, reversal agent use is rare in this population. Given the possible unmet need and subsequent patient recruitment challenges, designing pediatric clinical trials of reversal agents requires innovative approaches.

## Introduction

Venous thromboembolism (VTE) in children is uncommon, with recent estimates of 106 cases per 10,000 overall in hospitalized children [1]. It is most often associated with risk factors, such as cancer and its treatment, congenital heart disease, inflammatory/autoimmune diseases, prior history of VTE, severe trauma, and systemic hormonal contraceptive use [2–5]. VTE in hospitalized children is a serious event associated with acute and chronic complications, such as pulmonary embolism and cerebrovascular events, resulting in an increased risk of mortality [6,7]. Anticoagulant therapy is, therefore, often warranted to prevent and treat VTE; however, children taking anticoagulants are at risk of acute hemorrhage, which may be associated with high morbidity and mortality [8].

A small number of anticoagulants are licensed for use in pediatric patients [9–11] and recommended in guidelines, but supporting evidence is generally limited [12]. Optimal type, duration, and dosage of anticoagulants are often estimated based on extrapolation from adult guidelines and expert recommendations, owing to a lack of pediatric clinical trial data [13]. Until recently, the most commonly used anticoagulants in children were vitamin K antagonists (primarily warfarin), unfractionated heparin, and low-molecular-weight heparins (LMWH) [14]. LMWH requires subcutaneous injections, and warfarin requires multiple venipunctures to monitor the international normalized ratio [12,15]. Warfarin prescribing in children versus the adult population is further complicated by greater variability in the international normalized ratio and dosing requirements, which cause significant burden for patients and their caregivers. Dosing complexity and safety issues [16,17] highlight the need for alternative anticoagulants with a reduced burden on pediatric patients, such as direct oral anticoagulants (DOACs) [18].

DOACs are the preferred treatment option for VTE in adults because they are more effective in preventing VTE and carry a lower risk of major bleeding than warfarin [19,20]. These agents also do not require frequent dose adjustment, dietary restrictions, or monitoring, and therefore present a reduced burden to the patient and caregiver compared with warfarin and heparins [20]. Consequently, the use of DOACs has great potential to treat VTE in most situations where warfarin and/or LMWH have been used previously [14]. The factor Xa (FXa) inhibitor rivaroxaban and the direct thrombin inhibitor dabigatran were approved by the European Medicines Agency (EMA) in 2020 and the US Food and Drug Administration (FDA) in 2021 for the treatment and secondary prevention of VTE in pediatric patients [9,10,21,22]. Rivaroxaban is also approved by the FDA for primary thromboprophylaxis in children aged ≥2 years with congenital heart disease who have undergone the Fontan procedure [10,23]. The approval of rivaroxaban for these indications was based on results from 2 phase 3 studies: EINSTEIN-Jr, which demonstrated similar efficacy and safety with reduced thrombotic burden compared with standard anticoagulant therapy in children aged 0–17 years with acute VTE [24], and the UNIVERSE study, which showed a similar safety profile and numerically fewer thrombotic events with rivaroxaban versus aspirin in children who underwent a Fontan procedure [23]. Recent studies show promising efficacy and safety results for other FXa inhibitors, such as apixaban and edoxaban, in the pediatric population [25–27].

Although settings and treatment regimens varied, clinical trials with dabigatran and rivaroxaban have shown that rates of major bleeding events in pediatric patients (0%-2%) [24,25,27] are similar to those reported in adults (0.8%-2.8%) [28–30]. DOACs may present a lower risk of bleeding than heparins and warfarin [31], but reversal agents may still be necessary to manage bleeding in patients, such as those with life-threatening or uncontrolled bleeding and those who require emergency invasive procedures or surgery [32]. Andexanet alfa is a catalytically inert recombinant FXa variant. It received conditional approval for rivaroxaban and apixaban reversal in adults with life-threatening or uncontrolled bleeding from the FDA with orphan drug designation in May 2018 [33] and from the EMA in June 2019 [34]. Clinical trial data on the efficacy and safety of andexanet alfa are restricted to adults [35–37], and published data on its use in children are limited to a single pediatric case report [38].

Current guidelines recommend that idarucizumab should be considered in adults on dabigatran treatment, while andexanet alfa should be considered in adults on apixaban and rivaroxaban treatment as well as for those on edoxaban, although this is off-label in most countries [39]. Four-factor prothrombin complex concentrate is a hemostatic agent approved for rapid stabilization of patients receiving warfarin but is also used off-label in patients receiving other anticoagulants if specific reversal agents are not available [39,40]. However, limited data from the adult population suggest that 4-factor prothrombin complex concentrate may be less effective than a specific reversal agent, such as andexanet alfa [41–44].

The present retrospective study used real-world data to describe prescription trends for anticoagulants over time in pediatric populations, particularly for direct oral FXa inhibitors compared with conventional anticoagulants (LMWH or warfarin), in order to assess the potential study population for prospective clinical trials of andexanet alfa in children. Additionally, the frequency of relevant bleeding events (indicating the potential need for andexanet alfa) was quantified, along with actual use of andexanet alfa. Use of andexanet alfa was also compared with other reversal or stabilizing agents in pediatric patients on the day of surgery to investigate the potential need for acute reversal of FXa inhibition.

## Materials and methods

### Data sources

This study was conducted using 2 real-world data sources: the TriNetX Global Network (TriNetX, LLC, Cambridge, MA), a source of electronic medical records (EMRs); and Optum’s deidentified Clinformatics^®^ Data Mart Database (CDM; Optum^®^, Inc., Eden Prairie, MN), a health insurance claims dataset. Compliance statements for each database can be found in S1 Text.

TriNetX contains deidentified and aggregated EMRs from approximately 234 million individuals at participating health care organizations (HCOs) with inpatient and outpatient facilities in 23 countries, predominantly in the United States [45]. At the time of analysis (December 5, 2024), 190 HCOs contributed to TriNetX, of which most were in the United States.

CDM is a deidentified database of administrative health claims for members of a single provider of commercial and Medicare Advantage health plans. This database captured claims from inpatient and outpatient visits, as well as pharmacy coverage, from January 1, 2007, to December 31, 2023, for approximately 75 million individuals in the United States. Data were accessed and analyzed in November 2024. CDM was used as a confirmatory source to validate the findings obtained from TriNetX.

### Ethics statement

As this was an analysis of deidentified data compliant with guidance related to the Health Insurance Portability and Accountability Act (HIPAA) privacy rule [46], institutional review board (IRB) approval was not required. Per Title 45 of the Code of Federal Regulations (CFR), Part 46 [47], the data analysis in this study was exempt from IRB review as it was a retrospective analysis of existing data (hence no patient intervention or interaction), and no patient-identifiable information was included in the datasets.

TriNetX is compliant with HIPAA, the US federal law that protects the privacy and security of healthcare data. The network provides only deidentified data of EMRs with no identifiable patient information; thus, research studies using this network do not require IRBs for ethical approval. Data obtained from CDM were deidentified under the expert determination method consistent with HIPAA and managed according to Optum customer use data agreements (45 CFR 164.514(b)(1) and Guidance Regarding Methods for De‐identification of Protected Health Information in Accordance with the HIPAA Privacy Rule).

### Study design and population

This study identified patients <18 years of age who were prescribed anticoagulants in the TriNetX and CDM data sources. For TriNetX, patients who were <18 years of age at the time they received their prescription between January 1, 2007, and December 31, 2023, (to compare like-for-like with CDM) were included in analyses of prescription trends, which included age at prescription, in order to capture pediatric patients receiving anticoagulants during each specific year. The analyses of bleeding and andexanet alfa use in the TriNetX database included patients who were <18 years of age as of December 5, 2024, in order to show the most current patient population that would be eligible for a pediatric trial. For CDM, patients who were <18 years of age at the time they received an anticoagulant prescription were identified and included in the study. Anticoagulants were defined as: rivaroxaban, apixaban, edoxaban, dabigatran, dabigatran etexilate, warfarin, or LMWH (comprising enoxaparin, dalteparin, tinzaparin, ardeparin, or danaparoid). Drugs were identified using RxNorm codes in TriNetX and National Drug Codes (NDCs) in CDM (S1 Table).

### Analyses

TriNetX data analyses were conducted within the TriNetX Live analytics platform. TriNetX presents aggregated patient counts, where patient numbers are rounded up to the nearest 10. The minimum count is 10 for all non-zero counts. For example, 1–10 individual patients receiving a drug or surgery would be read out as 10 patients and 11–20 would be read out as 20. CDM contains patient-level data, so true patient numbers were recorded. Given the descriptive nature of the study, no inferential statistical comparisons were performed.

#### Anticoagulant prescription trends.

To analyze prescription trends over time, the number of patients <18 years of age at the time they received their prescription for an anticoagulant were stratified by type of anticoagulant and by year, from 2007 to 2023, in both TriNetX and CDM. The total number of pediatric patients who had a hospital visit in the TriNetX platform increased from 3.4 to 12.5 million between 2007 and 2023; therefore, prescription data were normalized to account for this increase over time. The proportion of patients with a prescription for each anticoagulant type was calculated as a percentage of the number of unique patients with any anticoagulant prescription for each year. Patients with >1 anticoagulant prescription were counted once for each anticoagulant type. As several patients received >1 anticoagulant class per year, percentages summed to > 100%.

#### FXa inhibitor prescription trends by age group.

The distribution and trends of pediatric patients prescribed FXa inhibitors from 2013 to 2023 and segmented by age group were analyzed. Patients were categorized into age groups based on their age at the time of prescription: < 1, 1–5, 6–11, and 12–17 years. All patients included in the analysis were <18 years of age at the time of receiving an FXa inhibitor prescription (apixaban, edoxaban, or rivaroxaban), in both TriNetX and CDM. Percentages were calculated to provide a clear view of the distribution of patients across different age groups and years. For each year and age group, the following information was recorded:

- Numerator: the number of pediatric patients *in each age group* who were prescribed an FXa inhibitor for a given year
- Denominator: the *total* number of pediatric patients who were prescribed an FXa inhibitor within that year
- Patients per year (%): Percentage of patients per year within each age group was calculated by dividing the number of patients in each age group by the total number of pediatric patients on FXa inhibitors for that year and multiplying by 100.

For the TriNetX data source, due to the aggregated nature of the data, the TriNetX database does not allow tracking or follow-up of individual patients over time. Thus, there was a potential for double counting as a patient could age into another age group within the same year. In contrast, double counting was not an issue with CDM data, as access to the individual patient data allowed for a different approach. To avoid double counting, the data represent the first time a patient had been prescribed an FXa inhibitor within a given year. Each patient was counted only once per year, based on their age at the time of the first prescription within that year, to ensure that patients were categorized into age groups without duplication. The number of pediatric patients taking FXa inhibitors before 2013 was too low to provide meaningful results when divided by age group. Consequently, only data from 2013 onward are included in the reported data for this analysis. For instance, in 2011, just 10 pediatric patients on FXa inhibitors were reported in TriNetX and 4 in CDM, and in 2012, only 100 patients were reported in TriNetX and 19 in CDM. Given that the TriNetX platform rounds patient numbers to the nearest 10, the reporting error was deemed too substantial.

In both the TriNetX and CDM data sources, analyses focused on comparing the trends and patterns among different age groups over time. Descriptive statistics were used to summarize the data, and trends were visualized by plotting the percentages for each age group from 2013 to 2023.

#### Relevant bleeding and andexanet alfa use.

The main clinical outcome evaluated in TriNetX was relevant bleeding after prescription of a FXa inhibitor (rivaroxaban, apixaban, or edoxaban); relevant bleeding was defined as bleeding events that would, under normal circumstances, result in consideration of either transfusion or use of a reversal intervention in cases of potentially reversible underlying causes. These relevant bleeds were identified using *International Classification of Diseases, 10th Revision* (ICD-10) codes (S2 Table). In-hospital mortality among these patients was also assessed.

The use of andexanet alfa on the day of a relevant bleed and separately on the day of surgery were evaluated in TriNetX between May 3, 2018, (the date of conditional FDA approval of andexanet alfa in adults) and December 5, 2024. Surgery was evaluated regardless of the type of encounter (i.e., inpatient, emergency, or outpatient) or diagnosis (e.g., bleeding). The use of other reversal or stabilizing agents ≥7 days on the day of a relevant bleed and on the day of surgery was also assessed in TriNetX. The use of andexanet alfa in children was also evaluated in CDM. Andexanet alfa use was defined using RxNorm, Healthcare Common Procedure Coding System (HCPCS), ICD-10, and NDCs, and surgery was identified using Current Procedural Terminology (CPT) and Systematized Nomenclature of Medicine - Clinical Terms (SNOMED) codes (S1 Table) [48–52]. The use of other reversal or stabilizing agents was defined using RxNorm, HCPCS, CPT, Anatomical Therapeutic Chemical, and ICD-10 Procedure Coding System codes (S3 Table).

## Results

### Patient population

**Fig 1:**
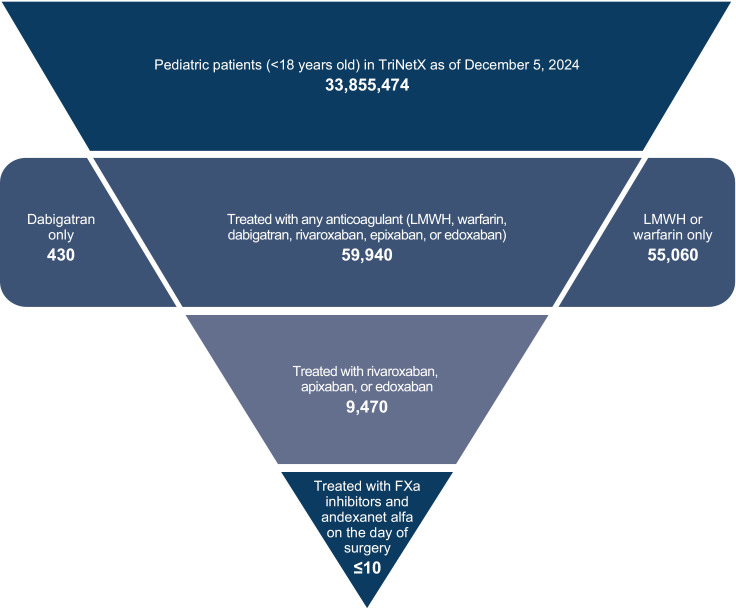
Patient Cohort in TriNetX, comprising patients currently <18 years of age as of December 2024. ^a^ FXA, Factor XA; LMWH, Low-molecular-weight heparin. Note that the TriNetX database rounds up to the nearest 10 patients. Anticoagulants were defined as standard anticoagulants (warfarin or lmwh [including enoxaparin, dalteparin, tinzaparin, ardeparin, or danaparoid]), FXA inhibitors (rivaroxaban, apixaban, or edoxaban), and dabigatran. ^a^ ≤ 10 patients were prescribed andexanet alfa on the day of a relevant bleed.

Of the > 33 million patients currently <18 years of age in the TriNetX Global Network as of December 5, 2024, 59,940 patients were prescribed standard anticoagulants (warfarin or LMWH), dabigatran, or FXa inhibitors (**Fig 1**). As this study focused on comparing prescription patterns across FXa inhibitors and standard anticoagulants, patients taking dabigatran were not included in further analyses; thus, the patient population included 59,780 patients. Overall, 73.8% of these patients were from the United States (S4 Table). The mean (standard deviation [SD]) age was 11.0 (5.0) years. The majority of patients (52.5%) were 12–17 years of age, 28.1% were 6–11 years of age, 19.1% were 1–5 years of age, and 1.3% were <1 year of age. A total of 54.8% of patients were male, 44.7% were female, and 0.5% were classified as unknown.

In the CDM data source, > 3.2 million US patients of any age received anticoagulants, and 6470 of those patients were <18 years of age at the time of receiving a prescription and were included in the study. The mean (SD) age was 12.5 (5.9) years. As observed in TriNetX, the majority of patients (68.6%) were 12–17 years of age, while 11.9% were 6–11 years of age, 10.6% were 1–5 years of age, and 8.8% were <1 year of age. A total of 54% of patients were male, and 46% were female.

### Anticoagulant prescribing trends

In the TriNetX data source, 92.1% of patients received warfarin or LMWH, and 15.8% received a direct oral FXa inhibitor (rivaroxaban, apixaban, or edoxaban; **Fig 1**). In the CDM data source, 88.3% of patients received warfarin or LMWH, and 16.8% received a direct oral FXa inhibitor. In both data sources, patients may have taken a direct oral FXa inhibitor and warfarin or LMWH.

**Fig 2:**
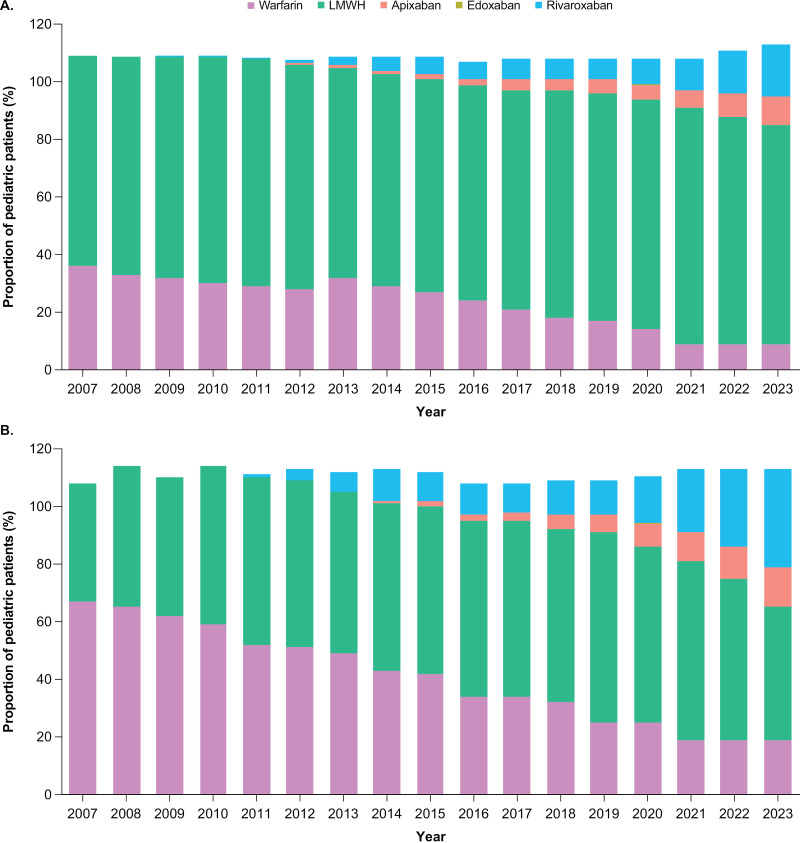
Anticoagulant prescribing trends among pediatric patients in TriNetX (A) and CDM (B) over time from 2007 until 2023. CDM, Clinformatics^®^ Data Mart Database; LMWH, low-molecular-weight heparin (includes danaparoid, tinzaparin, enoxaparin, dalteparin, and ardeparin). The data represent the proportion of patients <18 years old at the time of prescription who were being treated with each anticoagulant type, calculated as a percentage of the number of unique patients with any anticoagulant prescription, for each year. Some patients had prescriptions for > 1 anticoagulant type, so the proportions sum to over 100%. Note that TriNetX rounds values up to the nearest 10 patients.

From 2007 to 2008, all patients were prescribed either LMWH or warfarin in TriNetX. In 2007, 36.1% of pediatric patients with an anticoagulant prescription were prescribed warfarin and 72.9% were prescribed LMWH. High initial usage of warfarin was observed, with a steady decline over time to 18.1% in 2018 and down to 8.7% by 2023. LMWH showed consistent and high usage, with a gradual increase from 2007 (72.9%) that peaked in 2021 (82.0%) and slightly declined in 2023 (76.2%). FXa inhibitor usage, particularly rivaroxaban and apixaban, increased steadily over time. Rivaroxaban usage started in 2009 (albeit with very few patients, ≤ 10), with a gradual increase over time, reaching 18.0% by 2023. Similarly, apixaban was introduced in 2012 with minimal usage, increasing over time to 9.6% in 2023. Edoxaban usage remained low over time, starting from 2017, ranging from ≤10–30 patients and peaking in 2019 (**Fig 2A**). There was a significant increase in the total number of pediatric patients taking LMWH, warfarin, or FXa inhibitors over time, from 1440 in 2007, increasing to 8630 in 2014, and reaching 19,590 in 2023 (S1A Fig). Trends in the absolute number of pediatric patients prescribed each anticoagulant (warfarin, LMWH, rivaroxaban, apixaban, and edoxaban) were generally consistent with trends in the proportions of pediatric patients receiving these anticoagulants (S1A Fig).

**Fig 3:**
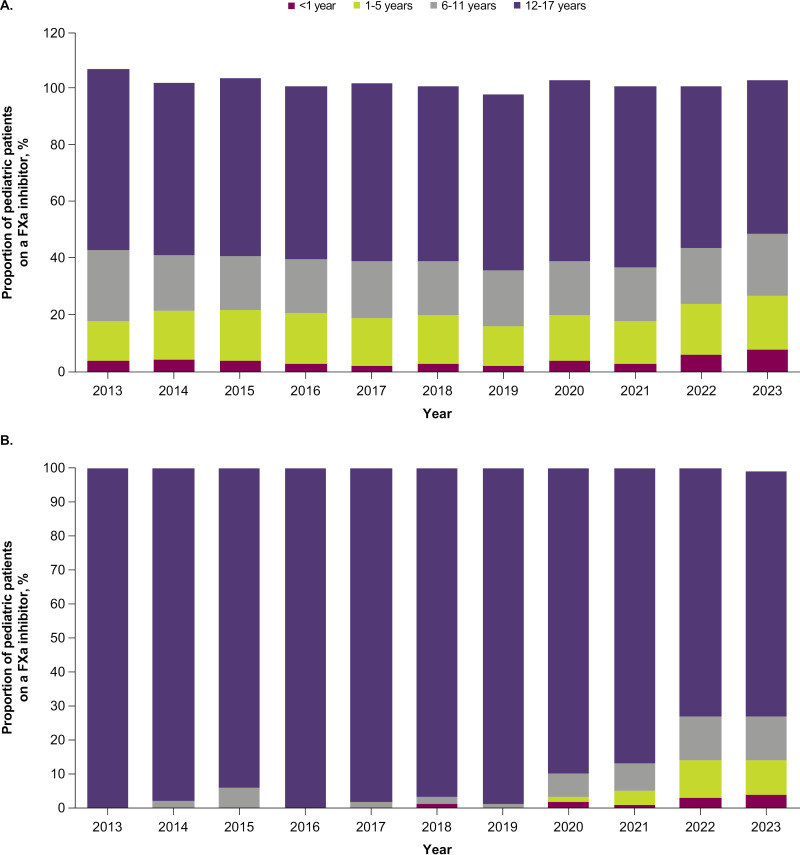
Trends in pediatric FXa inhibitor prescriptions by age group in TriNetX^a^ (A) and CDM (B) (2013 to 2023). CDM, Clinformatics^®^ Data Mart Database; FXa, factor Xa. Note that TriNetX rounds values up to the nearest 10 patients. For TriNetX, percentages may total >100% due to patient rounding and potential overlap between age groups.

The distribution and trends of pediatric patients prescribed FXa inhibitors were analyzed from 2013 through 2023 and segmented by age group (**Fig 3A**). There was a substantial increase in the total number of pediatric patients on FXa inhibitors from 2013 (280 patients) to 2023 (5260 patients), with noticeable trends in the distribution of these patients across different age groups. The number of patients in each age group was relatively low in the early years. For example, in 2013 the breakdown by age group was the following: < 1 year, n = ≤10; 1–5 years, n = 40; 6–11 years, n = 70; 12–17 years, n = 180. In contrast, in 2023, the number of patients in each age group was noticeably higher: < 1 year, n = 410; 1–5 years, n = 990; 6–11 years, n = 1140; 12–17 years, n = 2820. The 12- to 17-year-old age group consistently represented the highest percentage of pediatric patients on FXa inhibitors, starting at 64.3% in 2013, remaining largely stable around 61% to 64% up to 2021, and then decreasing to 53.6% by 2023. The 6- to 11-year-old age group remained somewhat stable, fluctuating between approximately 19% and 25% between 2013 and 2023. For the age group of 1- to 5-year-old patients, there was a general increase over time. In 2013, 14.3% of patients were 1–5 years old, with some fluctuation over time, reaching 18.8% by 2023. The < 1-year-old age group fluctuated between approximately 2% and 8% over time. Overall, while the 12- to 17-year-old age group consistently had the highest number of patients prescribed an FXa inhibitor, an increase in FXa inhibitor prescriptions in the < 1-year and 1- to 5-year-old age groups was observed in recent years.

In CDM, warfarin use was initially high; 67.1% of pediatric patients with an anticoagulant prescription in 2007 were prescribed warfarin. Warfarin prescriptions declined over time, down to 18.7% to 18.9% by 2021–2023 (**Fig 2B**). LMWH showed consistent usage, with only minor fluctuations, from 2013 to 2021, peaking at 65.8% in 2019 but declining to 45.6% by 2023. At the time of its introduction in 2011, rivaroxaban was prescribed to only 0.8% of pediatric patients with an anticoagulant prescription, but prescriptions increased steadily over time to 34.0% in 2023. A similar trend was observed for apixaban, which was introduced in 2014 and was prescribed to only 0.8% of pediatric patients with an anticoagulant prescription; apixaban prescriptions showed a gradual increase to 14.1% in 2023. Edoxaban was introduced in 2018, and usage remained minimal over time and was prescribed to < 1% of patients from 2018 to 2020, with no prescriptions 2021–2023. The number of unique pediatric patients with a prescription for any anticoagulant was more consistent year to year in the CDM than in the TriNetX data source. The number of unique pediatric patients with an anticoagulant prescription was 574 in 2007, peaked at 665 in 2021, and declined to 509 by 2023 (S1B Fig).

As observed with the TriNetX data, there was a noticeable increase in the number of pediatric patients prescribed an FXa inhibitor over time, from 2013 (34 patients) to 2023 (185 patients). The patient numbers were relatively low in 2013, particularly for the younger age groups. The 12- to 17-year-old age group consistently represented the highest percentage of pediatric patients on FXa inhibitors, starting at 100.0% in 2013, but showing a gradual decline from 2017 and a more pronounced decrease from 2020 onwards, reaching 72.4% in 2023 (**Fig 3B**). The 6- to 11-year-old age group was initially low and fluctuating but increased substantially from 6.7% in 2020 to approximately 13% in 2022 and 2023. The 1- to 5-year-old age group emerged in 2020 at 1.7% and showed a sharp increase to 10.3% in 2023. The percentage of patients in the < 1-year-old age group was consistently low, but showed an increasing trend in recent years, from 1.1% in 2018 to 4.3% in 2023. Overall, there was an increasing trend in the prescription of FXa inhibitors among the younger age groups over time in CDM, while the oldest age group (12–17 years) showed a decreasing trend. These trends are consistent with those observed in TriNetX (**Fig 3**).

Both data sources indicate a significant decline in warfarin usage over time, with a concurrent rise in the use of the newer anticoagulants rivaroxaban and apixaban, showing the evolution of anticoagulant therapy over time. LMWH maintained high usage rates in the pediatric population.

### Relevant bleeding events among patients receiving FXA inhibitors in TriNetX

**Fig 4:**
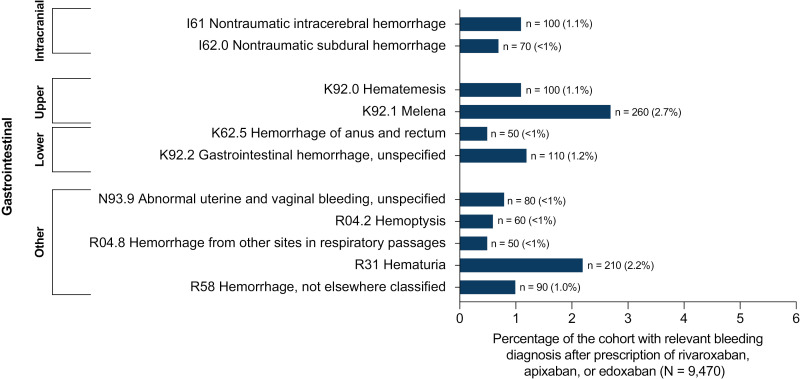
Most common relevant bleeding events among patients prescribed FXA inhibitors (N =  890). FXA, Factor XA; ICD-10, *International Classification Of Diseases, 10th revision*. A total of 890/9470 (9.4%) patients on FXA inhibitors experienced relevant bleeding events. only records comprising ≥0.5% of the total Cohort (N = 9470) are shown. All data are shown in S5 Table. ICD-10 codes were categorized as intracranial, gastrointestinal (lower and upper), or other. note that the TriNetX database rounds values up to the nearest 10 patients.

Of the 9470 pediatric patients in TriNetX who received rivaroxaban, apixaban, or edoxaban prescriptions, 890 (9.4%) patients had ≥ 1 relevant bleeding event that occurred ≥7 days after their first prescription. For patients taking LMWH (N = 51,330) and for those taking warfarin (N = 6330), rates of relevant bleeding were higher (14.1% [n = 7240] and 16.1% [n = 1020], respectively) than for those taking oral FXa inhibitors. Among patients taking an oral FXa inhibitor, melena was the most common type of bleed, affecting 2.7% of patients (n = 260), followed by hematuria (2.2% [n = 210]; **Fig 4** and S5 Table). Other bleeding events that occurred in ≤ 1.2% patients included unspecified gastrointestinal hemorrhages, nontraumatic intracerebral hemorrhage, hematemesis, and other hemorrhages. Of the 890 patients taking an oral FXa inhibitor who had a relevant bleed, 12.4% (n = 110) died in the hospital. The cause of death was not captured and may not have been directly related to the bleeding event.

### Andexanet alfa use

Of the 9120 pediatric patients who were prescribed a direct oral FXa inhibitor only between May 3, 2018, and December 3, 2024, in TriNetX, ≤ 10 patients (≤0.1%) were prescribed andexanet alfa on the same day they underwent surgery (**Fig 1**) and ≤ 10 (≤0.1%) patients were prescribed andexanet alfa on the day of a relevant bleed. In TriNetX, other reversal or stabilizing agents (S3 Table) were used in 7.8% (n = 710) of patients on the day of surgery and in 1.4% (n = 130) of patients on the day of a relevant bleed.

In CDM, no children received andexanet alfa

## Discussion

The landscape of anticoagulation in pediatric patients is evolving rapidly. The findings of our study, which is to our knowledge the most comprehensive study in terms of data for this population, suggest that the switch from conventional anticoagulants is gathering pace. Findings in this study from 2 large, real-world data sources (TriNetX and CDM) confirm a progressive shift in anticoagulant prescribing in children from 2007 to 2023, with trends indicating a decline in the use of warfarin and an increase in the use of DOACs, such as the FXa inhibitors rivaroxaban and apixaban. This shift likely represents a switch to agents with lower treatment and monitoring burden. Use of LMWH, which remains the primary option for a number of pediatric indications, generally remained high (TriNetX, ~ 73%-82%; CDM, ~ 41%-65%) throughout the study period, as expected, since LMWH often needs to be prescribed for ≥ 5 days for the initial anticoagulation prior to starting direct oral FXa inhibitor or warfarin treatment. Patient sample size for both TriNetX and CDM increased over time; thus, confidence in and accuracy of the data also increased. FXa inhibitors were most frequently prescribed in patients ≥12 years of age across both databases, likely because these patients could readily take oral tablets and pediatric formulations suitable for younger children were not available until 2021. As anticoagulant prescribing trends have changed, it is essential to understand key safety outcomes associated with anticoagulant use, particularly bleeding events, and the need for reversal agents to address those events.

Our data indicate that relevant bleeding events occurred in 9.4% of the pediatric population receiving FXa inhibitors, which was lower than the frequencies observed with LMWH (14.1%) or warfarin (16.1%) in the current study, but generally higher than those reported in randomized controlled trials or recent real-world studies of FXa inhibitors. In the phase 3 EINSTEIN-Jr trial, clinically relevant (major or nonmajor) bleeding was observed in 3% of patients treated with rivaroxaban and in 2% of patients who received standard anticoagulant therapy (heparin or vitamin K antagonist), while no cases of major bleeding were reported with rivaroxaban (compared with 1% with standard anticoagulant therapy). However, the majority of patients in that study had a relatively short treatment duration of 3 months [24]. In the SAXOPHONE trial of apixaban versus standard of care for thromboprophylaxis in children with cardiac disease, the rate of mild hematomas was 6.3% with apixaban and 1.6% with standard of care, and the rate of epistaxis was 15.9% and 9.7%, respectively [26]. In the ENNOBLE-ATE trial of edoxaban versus standard of care for thromboprophylaxis in pediatric cardiac patients, the rate of all bleeds (major, clinically relevant nonmajor, and minor) was 3.7% for edoxaban and 3.4% for standard of care [27]. Low rates of major or clinically relevant bleeds (<1%) were reported with edoxaban and apixaban in the ENNOBLE-ATE and SAXOPHONE trials, respectively [26,27]. Trials evaluating the use of dabigatran in pediatric patients have reported bleeding event rates (including major, clinically relevant nonmajor, and minor bleeding) of approximately 20% to 22%, with a low frequency of major bleeding events (1%-2%) [25,53]. In 2 other real-world database analyses, rates of major bleeding associated with rivaroxaban and apixaban in pediatric populations were also lower (<1%-3%) than in the current study [31,54]. One study included data from patients aged <18 years from 3 large US health claims databases, including CDM (which comprised 22% of the patient sample of that study), but only assessed bleeding outcomes through 2019 [31]. The other study assessed outcomes from 2015 to 2021 in patients aged ≤21 years at 15 specialized pediatric hemostasis centers in the United States, but included a relatively limited number of patients (n = 233) [54]. By contrast, a recent retrospective case series in pediatric cancer patients treated with rivaroxaban reported a higher rate of clinically relevant or major bleeding (20%) than in the phase 3 EINSTEIN-Jr trial (3%) [55]; however, the case series included children with thrombocytopenia or high bleeding risk, while the EINSTEIN-Jr trial excluded such patients [55]. Of note, the current study did not exclude any pediatric patients on the basis of their underlying diagnosis or any existing comorbidities. Further, the differences in major bleeding rates across these studies may have been related to differences in the definition of major bleeding, including more stringent criteria in clinical trials [26,27]. According to the FDA-approved prescribing information for both rivaroxaban and apixaban, these agents have a major bleeding rate of approximately 3% in adults [10,56], while a German registry study suggested bleeding rates as high as 6% in adults taking rivaroxaban [57].

Considering the high frequency of relevant bleeding found in our study, the use of andexanet alfa in children was found to be very rare. Out of > 33.0 million pediatric patients in TriNetX, 9470 were prescribed an FXa inhibitor, of whom ≤10 (≤0.1%) received andexanet alfa on the same day as surgery and ≤ 10 (≤0.1%) received andexanet alfa on the day of a relevant bleed. The rarity of andexanet alfa use was confirmed in data obtained from CDM, which showed no andexanet alfa use in pediatric patients. This could be due to the fact that andexanet alfa has been approved only for use in adults since 2018 [33,34] and any use in children is off-label as well as to the potential cost of treatment. The use of other reversal or stabilizing agents, including fibrinogen, prothrombin complex concentrate, and clotting factor concentrates (S3 Table), was also relatively low on the day of a relevant bleed (1.4%), while the rate was higher on the day of surgery (7.8%).

The recent rapid increase in rivaroxaban and apixaban prescribing in children may reflect their relative ease of use and lower bleeding risk compared with warfarin and LMWHs [24,26,27,53]. These agents are required to prevent or treat life-threatening VTE events, such as pulmonary embolism, especially in children with chronic diseases associated with increased thromboembolic risk, including congenital heart disease, inflammatory/autoimmune diseases, and cancer [4,18]. Anticoagulant prophylaxis is further required for indications such as indwelling central venous access devices or for thromboprophylaxis in pediatric trauma patients [12]. Anticoagulation in children requires an approach distinct from that used in adults due to inherent differences in the balance of hemostasis and thrombosis and response to anticoagulant therapy [58,59]. Further, the conditions that drive the need for anticoagulation also differ in pediatric patients compared to adults, for whom anticoagulants are typically prescribed for prevention of stroke or myocardial infarction and VTE associated with surgery.

The increasing use of anticoagulants in pediatric patients suggests a growing need for clinical development of novel anticoagulant reversal agents in order to balance the risks-benefits of anticoagulation in children, although development of these reversal agents is challenging for a number of reasons. Anticoagulant use is much less frequent in pediatric patients than in adult patients, and pediatric patients taking anticoagulants are spread across a wide geographic area, often far from a specialist center. Along with the relatively small available patient population, issues around the urgency of anticoagulant reversal and potential difficulties in obtaining appropriate consent from proxies also significantly complicate the mechanics of running a pediatric clinical trial of a reversal agent such as andexanet alfa. It is likely that such studies would require a large number of sites and take a considerable period of time to complete. Innovative study designs could help solve some of the difficulties of conducting trials of anticoagulant reversal agents in children. These could potentially include reduced comparator arms (e.g., usual care), or even use of an external comparison group to replace the comparator arm. It is crucial that a dialogue is established with regulators to optimize trial design to enable the development of reversal agents in order to provide patients with access to these crucial and potentially life-saving treatments [60–63].

There are a number of limitations associated with this study. As an observational, retrospective analysis, bias related to reporting, follow-up, and case selection is likely, but might have been partially mitigated by the separate evaluation of cohorts within each data source. Selection bias may exist in several areas, including the fact that patients in TriNetX had medical encounters within the contributing HCOs, most of which are large academic medical institutions in the United States. Some patients in this study may therefore have been enrolled in ongoing pediatric clinical trials of rivaroxaban and apixaban, biasing the population [23,24,64–69]. There was also the potential for reporting bias with respect to relevant bleeding events. Further, although the type of relevant bleeding reported would likely vary by age, an analysis of bleeding by age was beyond the scope of the current study. Data capture and representativeness may have changed over time, as suggested by the nearly 10-fold increase in the number of pediatric patients in the TriNetX platform from 2007 to 2023. In addition, in order to ensure privacy, data accessed on the TriNetX Live network is aggregated and does not contain individual-level patient records. This restriction allows for a broad reach but limits the level to which patient records can be analyzed. This rounding has the potential to impact the accuracy of calculations of FXa inhibitor–prescribing rates, FXa inhibitor–associated bleed rates, and andexanet alfa use, particularly as sample sizes get smaller. Furthermore, the percentages reported here are calculated based on rounded figures; thus, each category may not total 100% due to rounding. Further, the anticoagulant prescriptions identified in these analyses may not have represented the first lifetime prescription for patients, because any prior prescriptions not captured in TriNetX or CDM would not have been included in these analyses. Therefore, these data should be interpreted with caution in light of these potential limitations.

An additional limitation of the analyses presented here for the TriNetX data is that, due to the dynamic nature of the TriNetX Live network, a rerun of these analyses may not yield the exact same results. Further, we did not have access to health care providers’ notes; therefore, the reasons for surgery could not be determined, only the date of surgery and of reversal agent administration. Thus, although surgery served as a proxy for the need for FXa inhibitor reversal in the current study, relevant data to inform the need for reversal therapy is missing, including if the procedure was emergency surgery and the type of encounter. In addition, TriNetX records data on deaths that occur at the point of care at participating HCOs; however, deaths that took place outside the hospital setting (e.g., at home) are not well captured. Similarly, patient care received in other settings outside participating HCOs is also not captured. To address this limitation, the CDM, which includes patients treated across various settings, was used as a confirmatory source. There were, however, differences in the age distribution between TriNetX and CDM that may have limited comparability across the databases; the majority of patients in CDM were ≥12 years of age, while there was a more even distribution of ages from <1–17 years in TriNetX. This difference in age distribution may have stemmed from the difference in the data across the 2 databases; TriNetX only includes EMR data from participating hospitals, while CDM included data for inpatient, outpatient, and pharmacy claims.

An additional study limitation is that CDM includes data only for commercially insured and Medicare Advantage patients. These data lack information on uninsured or Medicaid populations, and the data are skewed toward a higher socioeconomic status (in this case, the parents of pediatric patients) than the general US population [70]. In contrast, TriNetX contains data for both insured and uninsured populations, which might mitigate the potential bias in CDM. Further, approximately 74% of patients identified in TriNetX and all patients in CDM were based in the United States, which may limit generalizability to other areas of the world. The overall generalizability of these findings needs validation in other populations and data sources. In addition, further analysis is required to address censoring, which was not taken into account in the current analysis.

In the coming years, the use of DOACs is likely to further increase in the pediatric population. A repeat snapshot of data in future years may well reveal more about the determinants of bleeding events and the need for anticoagulant reversal in this important and growing patient group. Larger patient numbers and a broader set of data across more databases may overcome some of the limitations of our research.

## Conclusions

This retrospective real-world data study highlights that increasing numbers of children and adolescents who require anticoagulants are now receiving direct oral FXa inhibitors instead of warfarin. This study represents an initial effort to define the size and burden of anticoagulant use in pediatric patients in this changing treatment landscape. The use of the FXa reversal agent andexanet alfa is very rare in pediatric patients. The lack of a pediatric approval for andexanet alfa represents a potential unmet medical need because of the potentially life-threatening nature of bleeding associated with FXa inhibitors.

## Supporting information

S1 Text Compliance statements. (DOCX)

S1 Table RxNorm, HCPCS, CPT, SNOMED, ICD-10, and NDC codes for anticoagulant and surgical treatments in TriNetX. CPT, Current Procedural Terminology; FXa, factor Xa; HCSPS, Healthcare Common Procedure Coding System; ICD-10, *International Classification of Diseases, 10th Revision*; NDC, National Drug Code; SNOMED, Systematized Nomenclature of Medicine - Clinical Terms.
(DOCX)

S2 Table ICD-10 codes to define relevant bleeding. ICD-10, *International Classification of Diseases, 10th Revision*; GI, gastrointestinal.
(DOCX)

S3 Table RxNorm, HCPCS, ICD-10-PCS, ATC, and CPT codes for defining reversal or stabilizing treatments beyond andexanet alfa in TriNetX. aHCSPS, Healthcare Common Procedure Coding System; ICD-10-PCS, *International Classification of Diseases, 10th Revision, Procedure Coding System*; ATC, Anatomical Therapeutic Chemical; CPT, Current Procedural Terminology; IU, international units. ^a^Not all of these reversal or stabilizing treatments have not been approved for treatment in pediatric patients.
(DOCX)

S4 Table Proportion of pediatric patients on anticoagulants per country identified in TriNetX. aCDM, Clinformatics^®^ Data Mart Database. ^a^In CDM, all patients were from the United States.
(DOCX)

S5 Table Number of patients diagnosed with a relevant bleeding event among those prescribed FXa inhibitors (n = 890). FXa, factor Xa; GI, gastrointestinal. ^a^Percentages calculated based on the total cohort (N = 9470).
(DOCX)

S1 Fig Absolute number of pediatric patients stratified by anticoagulant prescription from 2007 to 2023 in TriNetX (A) and CDM (B). LMWH, low-molecular-weight heparin (includes danaparoid, tinzaparin, enoxaparin, dalteparin, and ardeparin); CDM, Clinformatics^®^ Data Mart Database. Patient counts per anticoagulant therapy. Note that TriNetX rounds values up to the nearest 10 patients.
(DOCX)

## Abbreviations

CDM: Clinformatics® Data Mart Database
CPT: Current Procedural Terminology
DOAC: direct oral anticoagulant
EMA: European Medicines Agency
EMR: electronic medical record
FDA: US Food and Drug Administration
FXa: factor Xa
HCO: health care organization
HCPCS: Healthcare Common Procedure Coding System
HIPAA: Health Insurance Portability and Accountability Act
ICD-10: International Classification of Diseases, 10th Revision
IRB: institutional review board
LMWH: low-molecular-weight heparin
NDC: National Drug Codes
SD: standard deviation
SNOMED: Systematized Nomenclature of Medicine - Clinical Terms
VTE: venous thromboembolism
