# Supplementary material for: Anticoagulant prescribing trends, bleeding events, and reversal agent use in pediatric patients: A retrospective, real-world study
Source: PLoS One. 2025 May 8;20(5):e0323137. doi: 10.1371/journal.pone.0323137 (PMC12061172; doi:10.1371/journal.pone.0323137)
Supplement: S1 Text — (DOCX) [file pone.0323137.s001.docx]

**S1 Text. Compliance statements**

TriNetX compliance statement

TriNetX, LLC complies with the Health Insurance Portability and Accountability Act (HIPAA) and any additional data privacy regulations applicable to the contributing health care organizations. TriNetX is certified to the ISO 27001:2013 standard and maintains an Information Security Management System to protect the health care data to which it has access and meet the HIPAA Security Rule requirements. Any data displayed on the TriNetX Platform in aggregate form, or any patient-level data provided in a dataset generated by the TriNetX Platform, only contains deidentified data as per the deidentification standard defined in Section §164.514(a) of the HIPAA Privacy Rule. Because this study used only deidentified patient records and did not involve the collection, use, or transmittal of individually identifiable data, this study was exempted from Institutional Review Board approval.

Optum^®^ compliance statement

Optum’s deidentified Clinformatics^®^ Data Mart Database (CDM) is derived from a database of administrative health claims for members of large commercial and Medicare Advantage health plans. The database includes approximately 17 to 19 million annual covered lives, for a total of >65 million unique lives over a 15-year period (January 2007-October 2022). The CDM is statistically deidentified under the expert determination method (consistent with HIPAA) and managed according to CDM customer data use agreements. CDM administrative claims submitted for payment by providers and pharmacies are verified, adjudicated, and deidentified prior to inclusion. These data, including patient-level enrollment information, are derived from claims submitted for all medical and pharmacy health care services with information related to health care costs and resource utilization. The population is geographically diverse, spanning all 50 states.
