# Supplementary material for: Anticoagulant prescribing trends, bleeding events, and reversal agent use in pediatric patients: A retrospective, real-world study
Source: PLoS One. 2025 May 8;20(5):e0323137. doi: 10.1371/journal.pone.0323137 (PMC12061172; doi:10.1371/journal.pone.0323137)
Supplement: S1 Table — CPT, Current Procedural Terminology; FXa, factor Xa; HCSPS, Healthcare Common Procedure Coding System; ICD-10, International Classification of Diseases, 10th Revision; NDC, National Drug Code; SNOMED, Systematized Nomenclature of Medicine - Clinical Terms. (DOCX) [file pone.0323137.s002.docx]

**S1 Table. RxNorm, HCPCS, CPT, SNOMED, ICD-10, and NDC codes for anticoagulant and surgical treatments in TriNetX and CDM**

| **Treatment** | **Code** |
| --- | --- |
| **RxNorm** | |
| Rivaroxaban | 1114195 |
| Edoxaban | 1599538 |
| Apixaban | 1364430 |
| Dabigatran | 1546356 |
| Dabigatran etexilate | 1037042 |
| Danaparoid | 78484 |
| Tinzaparin | 69646 |
| Enoxaparin | 67108 |
| Dalteparin | 67109 |
| Ardeparin | 87866 |
| Warfarin | 11289 |
| Andexanet alfa | 2045114 |
| **HCSPS** | |
| Injection, coagulation FXa (recombinant) inactivated-zhzo (Andexxa), 10 mg | J7169 |
| **CPT** | |
| Surgery | 1003143 |
| **SNOMED** | |
| Surgical procedure | 387713003 |
| **ICD-10** | |
| Introduction of inactivated coagulation FXa (Andexxa) into peripheral vein, percutaneous approach, new technology group 2 | XW03372 |
| Introduction of inactivated coagulation FXa (Andexxa) into central vein, percutaneous approach, new technology group 2 | XW04372 |
| **NDC, rivaroxaban** | |
| Xarelto oral granule for reconstitution, 1 mg/mL | 50458057501 |
| Xarelto oral tablet, 2.5 mg | 50458057701  50458057710  50458057718  50458057760 |
| Xarelto oral tablet, 10 mg | 50458058010  50458058030  50458058090 |
| Xarelto oral tablet, 15 mg | 50458057810  50458057830  50458057890 |
| Xarelto oral tablet, 20 mg | 50458057910  50458057930  50458057989  50458057990 |
| Xarelto starter pack, oral kit, 15-20 mg | 50458058451 |
| **NDC, edoxaban** | |
| Savaysa oral tablet, 15 mg | 65597020130 |
| Savaysa oral tablet, 30 mg | 65597020205  65597020230  65597020290 |
| Savaysa oral tablet, 60 mg | 65597020305  65597020330  65597020390 |
| **NDC, apixaban** | |
| Eliquis oral tablet, 2.5 mg | 00003089321  00003089331 |
| Eliquis oral tablet, 5 mg | 00003089421  00003089431  00003089470 |
| Eliquis starter pack for treatment of DVT and PE oral tablets, 5 mg | 00003376474 |
| **NDC, enoxaparin** | |
| Lovenox injectable solution, 30 mg/0.3 mL | 00075062403  00075062430  00075062431  00075801301  00075801310 |
| Enoxaparin sodium injectable solution, 30 mg/0.3 mL | 00548560100  00548560100  00548563100  00703853021  00703853023  00781311963  00781313301  00781313363  00781323801  00781323863  00955100310  16714000601  16714000610  60505079100  60505079104  62037083920  63323053301  63323053313  63323053383  63323053393  63323055921  63323055963  63323055965  63323055993  63323056883  63323056894  68001045742  68001045743  70710175702  70710175706  71288041080  71288041081 |
| Lovenox injectable solution, 40 mg/0.4 mL | 00075062040  00075062041  00075801401  00075801410  54868544000  54868544001  58016487201  68115074910 |
| Enoxaparin sodium injectable solution, 40 mg/0.4 mL | 00548560200  00548563200  00703854021  00703854023  00781311964  00781322402  00781322464  00781324602  00781324664  00955100410  16714001601  16714001610  60505079200  60505079204  62037084920  63323053501  63323053508  63323053587  63323053598  63323056421  63323056497  63323056887  63323056896  68001045842  68001045843  70710175802  70710175806  71288041082  71288041083 |
| Lovenox injectable solution, 60 mg/0.6 mL | 00075062160  00075062161  00075801601  00075801610  54868558700  54868558701  68115077410 |
| Enoxaparin sodium injectable solution, 60 mg/0.6 mL | 00548560300  00548563300  00703856021  00703856023  00781311966  00781325603  00781325666  00781335603  00781335666  00955100610  16714002601  16714002610  60505079300  60505079304  62037086120  63323056621  63323056698  63323056888  63323056898  63323060701  63323060708  63323060788  63323060798  68001045942  68001045943  70710175902  70710175906  71288041084  71288041085 |
| Lovenox injectable solution, 80 mg/0.8 mL | 00075062280  00075062281  00075801801  00075801810  54868511200 |
| Enoxaparin sodium injectable solution, 80 mg/0.8 mL | 00548560400  00548563400  00703868021  00703868023  00781311968  00781326204  00781326268  00781342804  00781342868  00955100810  16714003601  16714003610  60505079400  60505079404  62037086220  63323053108  63323053190  63323053198  63323056890  63323056899  63323058421  63323058463  63323058465  63323058499  68001046042  68001046043  70710176006  71288041086  71288041087 |
| Lovenox injectable solution, 100 mg/mL | 00075062300  00075062301  00075802001  00075802010  54868583500 |
| Enoxaparin sodium injectable solution, 100 mg/mL | 00078135006  00548560500  00548563500  00703858021  00703858023  00781311969  00781326805  00781326869  00781350005  00781350069  00955101010  16714004601  16714004610  60505079501  60505079504  62037086320  63323056884  63323056895  63323058621  63323058696  63323060501  63323060504  63323060584  63323060594  68001046142  68001046143  70710176102  70710176106  71288041088  71288041089 |
| Lovenox injectable solution, 120 mg/0.8 mL | 00075291201  00075291202  00075802201  00075802210  54868583700 |
| Enoxaparin sodium injectable solution, 120 mg/0.8 mL | 00548560600  00548563600  00703861021  00703861023  00781312168  00781329804  00781329868  00781361204  00781361268  00955101210  16714005601  16714005610  60505079600  60505079604  62037086420  63323056990  63323056999  63323060901  63323060990  63323065521  63323065599  68001046242  68001046243  70710176002  70710176206  71288041180  71288041181 |
| Lovenox injectable solution, 150 mg/mL | 00075291501  00075291502  00075802501  00075802510 |
| Enoxaparin sodium injectable solution, 150 mg/mL | 00548560700  00548563700  00703851021  00703851023  00781312169  00781329905  00781329969  00781365505  00781365569  00955101510  16714006601  16714006610  60505079800  60505079804  62037086620  63323053701  63323053784  63323056984  63323056995  63323058921  63323058994  68001046342  68001046343  70710176302  70710176306  71288041182  71288041183 |
| Lovenox injectable solution, 300 mg/3 mL | 00075062603  00075062604  00075803001 |
| Enoxaparin sodium injectable solution, 300 mg/3 mL | 00548560800  00781312293  00955101601  63323053903  63323056586  63323056593  68001046441 |
| **NDC, warfarin** | |
| Coumadin oral tablet, 1 mg | 00056016901  00056016970  00056016975  00056016990  00247152800  00247152830  54569444300  54868212800  66267062900 |
| Jantoven oral tablet, 1 mg | 00832121100  00832121101  00832121110  00832121189 |
| Warfarin sodium oral tablet, 1 mg | 00093171201  00093171210  00182267101  00182267110  00182267189  00339653712  00378880101  00378880110  00406205201  00406205210  00555083102  00555083105  00615454729  00781035207  15330010001  15330010010  23490647801  23490647802  23490647803  31722032701  31722032710  43063047130  43063065530  51079090820  51407034101  51407034110  51407078401  51407078410  51672402701  51672402703  51672402707  52959092430  54569622500  54868434900  55289034030  55887026401  55887026430  55887026490  57237011901  57237011999  58864077315  59772035204  59772035207  59772035208  59772035270  60429078401  60429078410  60429078477  65162076110  65162076111  68080414677  68084014677  68115035930  68382005201  68382005210  76282032701  76282032710 |
| Coumadin oral tablet, 2 mg | 00056017001  00056017030  00056017070  00056017075  00056017090  00247152900  00247152930  54569015800  54569015801  54868212900  55289014397  66267063600  67544040115 |
| Jantoven oral tablet, 2 mg | 00832121200  00832121201  00832121210  00832121289 |
| Warfarin sodium oral tablet, 2 mg | 00093171301  00093171310  00182267201  00182267210  00182267289  00247225203  00247225206  00339653812  00378880201  00378880210  00406205301  00406205310  00555086902  00555086905  00615150929  00781036307  15330010101  15330010110  21695067330  23490648001  23490648002  23490648003  31722032801  31722032810  51079090920  51407034201  51407034210  51407078501  51407078510  51672402801  51672402803  51672402807  52959092530  54569622400  54868442200  54868442201  54868442203  55045290200  55887092630  55887092690  57237012001  57237012099  58864087930  59772036304  59772036307  59772036308  59772036370  60429078501  60429078510  60429078577  60429078590  62584098401  62584098411  62584098477  65162076210  65162076211  66336025030  68115039930  68382005301  68382005310  71610046230  71610057430  71610057435  71610057440  71610057445  71610057453  71610057460  76282032801  76282032810 |
| Coumadin oral tablet, 2.5 mg | 00056017601  00056017630  00056017670  00056017675  00056017690  00247153000  00247153030  49999041130  54569021201  54868215400  66267063000 |
| Jantoven oral tablet, 2.5 mg | 00832121300  00832121301  00832121310  00832121389 |
| Warfarin sodium oral tablet, 2.5 mg | 00093171401  00093171410  00182267301  00182267310  00182267389  00182276301  00339653912  00378882501  00378882510  00406206401  00406206410  00406506410  00555083202  00555083205  00615151029  00781036407  15330010201  15330010210  23490648101  23490648102  23490648103  31722032901  31722032910  51079091020  51407034301  51407034310  51407078601  51407078610  51672402901  51672402903  51672402907  54569586800  54868440000  55887057710  55887057730  55887057760  55887057790  57237012101  57237012199  58864003530  59772036404  59772036407  59772036408  59772036470  60429078601  60429078610  60429078645  60429078677  62584098601  63739036101  63739036103  63739036110  63739036115  65162076310  65162076311  68080402777  68084002701  68084002711  68084402777  68382006401  68382006410  71610059330  76282032901  76282032910 |
| Coumadin oral tablet, 3 mg | 00056018801  00056018870  00056018875  00056018890  66267063100 |
| Jantoven oral tablet, 3 mg | 00832121400  00832121401  00832121410  00832121489 |
| Warfarin sodium oral tablet, 3 mg | 00093171501  00182267401  00182267489  00339654012  00378880301  00378880310  00406205401  00406205410  00555092502  00615454829  00781036607  15330026601  31722033001  31722033010  51407034401  51407034410  51407078701  51407078710  51672403001  51672403003  51672403007  54868487100  55887004401  55887004430  55887004460  55887004490  57237012201  57237012299  59772036607  60429078701  60429078710  60429078745  60429078777  63739036201  63739036203  63739036210  63739036215  65162076410  65162076411  68084414777  68382005401  68382005410  76282033001  76282033010 |
| Coumadin oral tablet, 4 mg | 00056016801  00056016870  00056016875  00056016890  00247153100  00247153130  54868339900  66267063200 |
| Jantoven oral tablet, 4 mg | 00832121500  00832121501  00832121510  00832121589  54868082500 |
| Warfarin sodium oral tablet, 4 mg | 00093171601  00093171610  00182267501  00182267589  00339654112  00378880401  00378880410  00406205501  00406205510  00555087402  00555087405  00615454929  00781036907  15330026701  23490648201  23490648202  23490648203  31722033101  31722033110  43063021830  49999092310  51407034501  51407034510  51407078801  51407078810  51672403101  51672403103  51672403107  54569586900  54868440200  55887046430  55887046490  57237012301  57237012399  58016008300  58016008330  58016008360  58016008390  59772036907  59772036908  59772036970  60429078801  60429078810  60429078830  60429078877  63739036301  63739036303  63739036310  63739036315  65162076510  65162076511  68080414877  68382005501  68382005510  71610058930  76282033101  76282033110 |
| Coumadin oral tablet, 5 mg | 00056017201  00056017230  00056017270  00056017275  00056017290  00247153200  00247153230  49999009330  54569015900  54569015901  54569854200  54868125900  55289028630  55289028650  55289028697  58864022314  66267063300 |
| Jantoven oral tablet, 5 mg | 00832121600  00832121601  00832121610  00832121689  54868520700 |
| Warfarin sodium oral tablet, 5 mg | 00093172101  00093172110  00182267601  00182267610  00182267689  00339654212  00378880501  00406205601  00406205610  00555083302  00555083305  00615151229  00781037707  15330026801  15330026810  21695067730  23490648301  23490648302  23490648303  31722033201  31722033210  43063017614  43063017630  49999057600  49999057610  49999057620  51079091320  51407034601  51407034610  51407078901  51407078910  51672403201  51672403203  51672403207  52959092630  54569493400  54569493401  54569493402  54868428600  55045288108  55289077314  55289077330  55289077360  55289077390  55887057810  55887057830  55887057860  55887057886  55887057890  57237012401  57237012499  58864069814  59772037704  59772037707  59772037708  59772037770  60429078901  60429078910  60429078915  60429078930  60429078940  60429078945  60429078977  60429078990  62580499477  62584099401  62584099411  62584099477  63739036401  63739036403  63739036410  63739036415  65162076610  65162076611  66336025230  68115065900  68382005601  68382005610  68382005616  71610057515  71610057520  71610057525  71610057530  71610057535  71610057538  71610057540  71610057545  71610057550  71610057553  71610057555  71610057560  76282033201  76282033210 |
| Coumadin intravenous fluid for injection, 5 mg | 00059032435  00590032435 |
| Coumadin oral tablet, 6 mg | 00056018901  00056018970  00056018975  00056018990  54868525500  66267063400 |
| Jantoven oral tablet, 6 mg | 00832121700  00832121701  00832121710  00832121789  54868121600 |
| Warfarin sodium oral tablet, 6 mg | 00093171801  00182267701  00182267789  00339654312  00378880601  00378880610  00406205701  00555092602  00615455029  00781038107  15330010601  31722033301  51407034701  51407034710  51407079001  51407079010  51672403301  51672403303  54569631200  54868487300  57237012501  57237012599  59772038107  60429079001  60429079010  60429079015  60429079077  65162076710  65162076711  68382005701  76282033301  76282033310 |
| Coumadin oral tablet, 7.5 mg | 00056017301  00056017370  00056017375  00247153300  00247153330  66267063500 |
| Jantoven oral tablet, 7.5 mg | 00832121800  00832121801  00832121850  00832121889 |
| Warfarin sodium oral tablet, 7.5 mg | 00093171901  00093172301  00182267801  00182267889  00339654412  00378887501  00378887510  00406205801  00555083402  00555083405  00615455129  00781038607  15330010701  23490648401  23490648402  23490648403  31722033401  49999082900  51079091520  51407034801  51407079101  51672403401  51672403403  54569631300  54868495000  57237012601  57237012699  59772038607  59772038670  60429079101  60429079177  65162076810  65162076811  68382005801  71610051930  76282033401 |
| Coumadin oral tablet, 10 mg | 00056017401  00056017470  00056017475  00247153400  00247153430  66267062800 |
| Jantoven oral tablet, 10 mg | 00832121900  00832121901  00832121950  00832121989 |
| Warfarin sodium oral tablet, 10 mg | 00093172001  00182267901  00182267989  00339654512  00378881001  00378881010  00406205901  00555083502  00555083504  00781038707  15330010801  31722033501  51407034901  51407079201  51672403501  51672403503  54868245401  54868245402  54868525800  57237012701  57237012799  58016069700  58016069730  58016069760  58016069790  59772038707  59772038770  60429079201  60429079245  60429079277  65162076910  65162076911  68382005901  71610045830  76282033501 |
| Warfarin sodium compounding powder, unspecified | 38779047410  38779047425  49452813601  49452813602 |
| Lovenox injectable solution, 30 mg/0.3 mL | 00075062403  00075062430  00075062431  00075801301  00075801310 |
| Enoxaparin sodium injectable solution, 30 mg/0.3 mL | 00548560100  00548563100  00703853021  00703853023  00781311963  00781313301  00781313363  00781323801  00781323863  00955100310  16714000601  16714000610  60505079100  60505079104  62037083920  63323053301  63323053313  63323053383  63323053393  63323055921  63323055963  63323055965  63323055993  63323056883  63323056894  68001045742  68001045743  70710175702  70710175706  71288041080  71288041081 |
| Lovenox injectable solution, 40 mg/0.4 mL | 00075062040  00075062041  00075801401  00075801410  54868544000  54868544001  58016487201  68115074910 |
| Enoxaparin sodium injectable solution, 40 mg/0.4 mL | 00548560200  00548563200  00703854021  00703854023  00781311964  00781322402  00781322464  00781324602  00781324664  00955100410  16714001601  16714001610  60505079200  60505079204  62037084920  63323053501  63323053508  63323053587  63323053598  63323056421  63323056497  63323056887  63323056896  68001045842  68001045843  70710175802  70710175806  71288041082  71288041083 |
| Lovenox injectable solution, 60 mg/0.6 mL | 00075062160  00075062161  00075801601  00075801610  54868558700  54868558701  68115077410 |
| Enoxaparin sodium injectable solution, 60 mg/0.6 mL | 00548560300  00548563300  00703856021  00703856023  00781311966  00781325603  00781325666  00781335603  00781335666  00955100610  16714002601  16714002610  60505079300  60505079304  62037086120  63323056621  63323056698  63323056888  63323056898  63323060701  63323060708  63323060788  63323060798  68001045942  68001045943  70710175902  70710175906  71288041084  71288041085 |
| Lovenox injectable solution, 80 mg/0.8 mL | 00075062280  00075062281  00075801801  00075801810  54868511200 |
| Enoxaparin sodium injectable solution, 80 mg/0.8 mL | 00548560400  00548563400  00703868021  00703868023  00781311968  00781326204  00781326268  00781342804  00781342868  00955100810  16714003601  16714003610  60505079400  60505079404  62037086220  63323053108  63323053190  63323053198  63323056890  63323056899  63323058421  63323058463  63323058465  63323058499  68001046042  68001046043  70710176006  71288041086  71288041087 |
| Lovenox injectable solution, 100 mg/mL | 00075062300  00075062301  00075802001  00075802010  54868583500 |
| Enoxaparin sodium injectable solution, 100 mg/mL | 00078135006  00548560500  00548563500  00703858021  00703858023  00781311969  00781326805  00781326869  00781350005  00781350069  00955101010  16714004601  16714004610  60505079501  60505079504  62037086320  63323056884  63323056895  63323058621  63323058696  63323060501  63323060504  63323060584  63323060594  68001046142  68001046143  70710176102  70710176106  71288041088  71288041089 |
| Lovenox injectable solution, 120 mg/0.8 mL | 00075291201  00075291202  00075802201  00075802210  54868583700 |
| Enoxaparin sodium injectable solution, 120 mg/0.8 mL | 00548560600  00548563600  00703861021  00703861023  00781312168  00781329804  00781329868  00781361204  00781361268  00955101210  16714005601  16714005610  60505079600  60505079604  62037086420  63323056990  63323056999  63323060901  63323060990  63323065521  63323065599  68001046242  68001046243  70710176002  70710176206  71288041180  71288041181 |
| Lovenox injectable solution, 150 mg/mL | 00075291501  00075291502  00075802501  00075802510 |
| Enoxaparin sodium injectable solution, 150 mg/mL | 00548560700  00548563700  00703851021  00703851023  00781312169  00781329905  00781329969  00781365505  00781365569  00955101510  16714006601  16714006610  60505079800  60505079804  62037086620  63323053701  63323053784  63323056984  63323056995  63323058921  63323058994  68001046342  68001046343  70710176302  70710176306  71288041182  71288041183 |
| Lovenox injectable solution, 300 mg/3 mL | 00075062603  00075062604  00075803001 |
| Enoxaparin sodium injectable solution, 300 mg/3 mL | 00548560800  00781312293  00955101601  63323053903  63323056586  63323056593  68001046441 |
| Orgaran subcutaneous solution, 750 anti-Xa units/0.6 mL | 00052083011  00052083061 |
| Fragmin subcutaneous solution, 2500 intl units/0.2 mL | 00013240691  00069019501  00069019502  62856025001  62856025010 |
| Fragmin subcutaneous solution, 5000 intl units/0.2 mL | 00013242691  00069019601  00069019602  62856050001  62856050010 |
| Normiflo subcutaneous solution, 5000 units/0.5 mL | 00008086001 |
| Fragmin subcutaneous solution, 7500 intl units/0.3 mL | 00013242601  00069020601  00069020602  62856075001  62856075010 |
| Fragmin subcutaneous solution, 10,000 intl units/mL | 00013243606  00013519001  00069021701  00069021702  62856010101  62856010110  62856010201 |
| Normiflo subcutaneous solution, 10,000 intl units/0.5 mL | 00008086101 |
| Fragmin subcutaneous solution, 12,500 intl units/0.5 mL | 00069022001  00069022002  62856012501  62856012510 |
| Fragmin subcutaneous solution, 15,000 intl units/0.6 mL | 00069022301  00069022302  62856015001  62856015010 |
| Fragmin subcutaneous solution, 18,000 intl units/0.72 mL | 00069022801  00069022802  62856018001  62856018010 |
| Innohep subcutaneous solution, 20,000 anti-FXa intl units/mL | 00056034208  00056034253  50222034208  54653034208  54653034253  67211034208  67211034253 |
| Fragmin subcutaneous solution, 25,000 intl units/mL | 00013519101  00069023201  62856025101 |
| **NDC, Andexxa** | |
| Andexxa recombinant, inactivated intravenous powder for injection, 100 mg | 69853010101 |
| Andexxa recombinant, inactivated intravenous powder for injection, 200 mg | 69853010201 |

CPT, Current Procedural Terminology; FXa, factor Xa; HCSPS, Healthcare Common Procedure Coding System; ICD-10, *International Classification of Diseases, 10th Revision*; NDC, National Drug Code; SNOMED, Systematized Nomenclature of Medicine-Clinical Terms.
