# Supplementary material for: Anticoagulant prescribing trends, bleeding events, and reversal agent use in pediatric patients: A retrospective, real-world study
Source: PLoS One. 2025 May 8;20(5):e0323137. doi: 10.1371/journal.pone.0323137 (PMC12061172; doi:10.1371/journal.pone.0323137)
Supplement: S2 Table — ICD-10, International Classification of Diseases, 10th Revision; GI, gastrointestinal. (DOCX) [file pone.0323137.s003.docx]

**S2 Table. ICD-10 codes to define relevant bleeding**

| **Type** | **ICD-10 code** | **Description** |
| --- | --- | --- |
| Intracranial | I60 | Nontraumatic subarachnoid hemorrhage |
|  | I61 | Nontraumatic intracerebral hemorrhage |
|  | I62.0 | Nontraumatic subdural hemorrhage |
|  | I62.1 | Nontraumatic extradural hemorrhage |
|  | I62.9 | Nontraumatic intracranial hemorrhage, unspecified |
| Upper GI | K92.0 | Hematemesis |
|  | K92.1 | Melena |
|  | I85.01 | Esophageal varices with bleeding |
|  | K22.11 | Ulcer of esophagus with bleeding |
|  | K25.0 | Acute gastric ulcer with hemorrhage |
|  | K25.2 | Acute gastric ulcer with both hemorrhage and perforation |
|  | K25.4 | Chronic or unspecified gastric ulcer with hemorrhage |
|  | K25.6 | Chronic or unspecified gastric ulcer with both hemorrhage and perforation |
|  | K26.0 | Acute duodenal ulcer with hemorrhage |
|  | K26.2 | Acute duodenal ulcer with both hemorrhage and perforation |
|  | K26.4 | Chronic or unspecified duodenal ulcer with hemorrhage |
|  | K26.6 | Chronic or unspecified duodenal ulcer with both hemorrhage and perforation |
|  | K27.0 | Acute peptic ulcer, site unspecified, with hemorrhage |
|  | K27.2 | Acute peptic ulcer, site unspecified, with both hemorrhage and perforation |
|  | K27.4 | Chronic or unspecified peptic ulcer, site unspecified, with hemorrhage |
|  | K27.6 | Chronic or unspecified peptic ulcer, site unspecified, with both hemorrhage and perforation |
|  | K28.0 | Acute gastrojejunal ulcer with hemorrhage |
|  | K28.2 | Acute gastrojejunal ulcer with both hemorrhage and perforation |
|  | K28.4 | Chronic or unspecified gastrojejunal ulcer with hemorrhage |
|  | K28.6 | Chronic or unspecified gastrojejunal ulcer with both hemorrhage and perforation |
|  | K29.01 | Acute gastritis with bleeding |
|  | K31.811 | Angiodysplasia of stomach and duodenum with bleeding |
|  | K31.82 | Dieulafoy lesion (hemorrhagic) of stomach and duodenum |
|  | K55.21 | Angiodysplasia of colon with hemorrhage |
|  | K62.5 | Hemorrhage of anus and rectum |
|  | K92.2 | Gastrointestinal hemorrhage, unspecified |
|  | N02.0 | Recurrent and persistent hematuria with minor glomerular abnormality |
| Lower GI | N02.1 | Recurrent and persistent hematuria with focal and segmental glomerular lesions |
|  | N02.2 | Recurrent and persistent hematuria with diffuse membranous glomerulonephritis |
|  | N02.3 | Recurrent and persistent hematuria with diffuse mesangial proliferative glomerulonephritis |
| Other bleeding | N02.4 | Recurrent and persistent hematuria with diffuse endocapillary proliferative glomerulonephritis |
|  | N02.5 | Recurrent and persistent hematuria with diffuse mesangiocapillary glomerulonephritis |
|  | N02.6 | Recurrent and persistent hematuria with dense deposit disease |
|  | N02.7 | Recurrent and persistent hematuria with diffuse crescentic glomerulonephritis |
|  | N02.8 | Recurrent and persistent hematuria with other morphologic changes |
|  | N02.9 | Recurrent and persistent hematuria with unspecified morphologic changes |
|  | N93.8 | Other specified abnormal uterine and vaginal bleeding |
|  | N93.9 | Abnormal uterine and vaginal bleeding, unspecified |
|  | N95.0 | Postmenopausal bleeding |
|  | M25.0 | Hemarthrosis |
|  | K66.1 | Hemoperitoneum |
|  | R04.1 | Hemorrhage from throat |
|  | R04.2 | Hemoptysis |
|  | R04.8 | Hemorrhage from other sites in respiratory passages |
|  | R04.9 | Hemorrhage from respiratory passages, unspecified |
|  | R31.0 | Gross hematuria |
|  | R31.1 | Benign essential microscopic hematuria |
|  | R31.2 | Other microscopic hematuria |
|  | R31.9 | Hematuria, unspecified |
|  | R58 | Hemorrhage, not elsewhere classified |
|  | 599.7 | Hematuria, unspecified |
|  | D68.3 | Hemorrhagic disorder due to circulating anticoagulants |
|  | H35.6 | Retinal hemorrhage |
|  | H43.1 | Vitreous hemorrhage |

ICD-10, *International Classification of Diseases, 10th Revision*; GI, gastrointestinal.

**References (S2 Table)**

[1] M. Jun, M.T. James, B.J. Manns, R.R. Quinn, P. Ravani, M. Tonelli, V. Perkovic, W.C. Winkelmayer, Z. Ma, B.R. Hemmelgarn, Alberta Kidney Disease Network, The association between kidney function and major bleeding in older adults with atrial fibrillation starting warfarin treatment: population based observational study, BMJ 350 (2015) h246.

[2] A. Abdul Sultan, J. West, O. Stephansson, M.J. Grainge, L.J. Tata, K.M. Fleming, D. Humes, J.F. Ludvigsson, Defining venous thromboembolism and measuring its incidence using Swedish health registries: a nationwide pregnancy cohort study, BMJ Open 5 (2015) e008864.

[3] L. Öhman, M. Johansson, J.H. Jansson, M. Lind, L. Johansson, Positive predictive value and misclassification of diagnosis of pulmonary embolism and deep vein thrombosis in Swedish patient registries, Clin. Epidemiol. 10 (2018) 1215–1221.

[4] S.D. Østergaard, M. Schmidt, E. Horváth-Puhó, R.W. Thomsen, H.T. Sørensen, Thromboembolism and the Oxford–AstraZeneca COVID-19 vaccine: side-effect or coincidence?, Lancet 397 (2021) 1441–1443.

[5] The Dudley Group NHS Foundation Trust, Freedom of information request FOI/013640 - venous thromboembolism, 2017. <https://www.dgft.nhs.uk/wp-content/uploads/2018/03/013640.pdf>. (accessed September 29, 2022).
