# Supplementary material for: Anticoagulant prescribing trends, bleeding events, and reversal agent use in pediatric patients: A retrospective, real-world study
Source: PLoS One. 2025 May 8;20(5):e0323137. doi: 10.1371/journal.pone.0323137 (PMC12061172; doi:10.1371/journal.pone.0323137)
Supplement: S3 Table — aHCSPS, Healthcare Common Procedure Coding System; ICD-10-PCS, International Classification of Diseases, 10th Revision, Procedure Coding System; ATC, Anatomical Therapeutic Chemical; CPT, Current Procedural Terminology; IU, international units. aNot all of these reversal or stabilizing treatments have not been approved for treatment in pediatric patients. (DOCX) [file pone.0323137.s004.docx]

**S3 Table. RxNorm, HCPCS, ICD-10-PCS, ATC, and CPT codes for defining reversal or stabilizing treatments beyond andexanet alfa in TriNetX^a^**

| **Treatment** | **Code** |
| --- | --- |
| **RxNorm** | |
| Prothrombin | 1441688 |
| Factor X | 4262 |
| Factor VII | 4254 |
| Protein C | 8834 |
| Factor IX | 4249 |
| Protein S | 34822 |
| Factor VIIa | 4256 |
| Fibrinogen | 4385 |
| Fibrinogen, I-125 | 314623 |
| **HCSPS** | |
| Prothrombin complex concentrate (human), Kcentra, per IU of factor IX activity | J7168 |
| Factor VIIa (antihemophilic factor, recombinant), NovoSeven RT, 1 µg | J7189 |
| Injection, human fibrinogen concentrate, not otherwise specified, 1 mg | J7178 |
| Injection, human fibrinogen concentrate, Fibryga, 1 mg | J7177 |
| Cryoprecipitated fibrinogen complex, pathogen reduced, each unit | P9026 |
| **ICD-10-PCS** | |
| Transfusion of nonautologous 4-factor prothrombin complex concentrate into vein, percutaneous approach | 30283B1 |
| Transfusion of nonautologous fibrinogen into peripheral vein, percutaneous approach | 30233T1 |
| Transfusion of nonautologous fibrinogen into central vein, percutaneous approach | 30243T1 |
| Transfusion of nonautologous pathogen-reduced cryoprecipitated fibrinogen complex into peripheral vein, percutaneous approach | 30233D1 |
| Transfusion of nonautologous pathogen-reduced cryoprecipitated fibrinogen complex into central vein, percutaneous approach | 30243D1 |
| Transfusion of nonautologous fibrinogen into peripheral vein, percutaneous approach | 30233T0 |
| Transfusion of autologous fibrinogen into central vein, percutaneous approach | 30243T0 |
| **CPT** | |
| Fibrinogen | 1011824 |
| **ATC** | |
| Fibrinogen | B02BB |

HCSPS, Healthcare Common Procedure Coding System; ICD-10-PCS, *International Classification of Diseases, 10th Revision, Procedure Coding System*; ATC, Anatomical Therapeutic Chemical; CPT, Current Procedural Terminology; IU, international units.

^a^Not all of these reversal or stabilizing treatments have not been approved for treatment in pediatric patients.
