# Supplementary material for: Anticoagulant prescribing trends, bleeding events, and reversal agent use in pediatric patients: A retrospective, real-world study
Source: PLoS One. 2025 May 8;20(5):e0323137. doi: 10.1371/journal.pone.0323137 (PMC12061172; doi:10.1371/journal.pone.0323137)
Supplement: S4 Table — aCDM, Clinformatics® Data Mart Database. aIn CDM, all patients were from the United States. (DOCX) [file pone.0323137.s005.docx]

**S4 Table. Proportion of pediatric patients on anticoagulants per country identified in TriNetX^a^**

| **Country** | **Patients, %** |
| --- | --- |
| United States | 73.8 |
| Germany | 6.7 |
| Brazil | 6.5 |
| Spain | 5.6 |
| Poland | 1.6 |
| United Kingdom | 1.6 |
| Belgium | 0.9 |
| Malaysia | 0.8 |
| Japan | 0.5 |
| India | 0.5 |
| Israel | 0.4 |
| United Arab Emirates | 0.3 |
| Australia | 0.2 |
| Colombia | 0.2 |
| Italy | 0.2 |
| Georgia | 0.2 |
| Ghana | 0.1 |
| Taiwan | 0.1 |
| Lithuania | 0.02 |

CDM, Clinformatics^®^ Data Mart Database.

^a^In CDM, all patients were from the United States.
