# Supplementary material for: Anticoagulant prescribing trends, bleeding events, and reversal agent use in pediatric patients: A retrospective, real-world study
Source: PLoS One. 2025 May 8;20(5):e0323137. doi: 10.1371/journal.pone.0323137 (PMC12061172; doi:10.1371/journal.pone.0323137)
Supplement: S5 Table — FXa, factor Xa; GI, gastrointestinal. aPercentages calculated based on the total cohort (N = 9470). (DOCX) [file pone.0323137.s006.docx]

**S5 Table. Number of patients diagnosed with a relevant bleeding event among those prescribed FXa inhibitors (n = 890)**

| **Type** | **Specific ICD-10 code** | | **On rivaroxaban, apixaban, or edoxaban, n (%)^a^** |
| --- | --- | --- | --- |
| Intracranial | I60 | Nontraumatic subarachnoid hemorrhage | 40 (0.4) |
|  | I61 | Nontraumatic intracerebral hemorrhage | 100 (1.1) |
|  | I62.0 | Nontraumatic subdural hemorrhage | 70 (0.7) |
|  | I62.1 | Nontraumatic extradural hemorrhage | 10 (0.1) |
|  | I62.9 | Nontraumatic intracranial hemorrhage, unspecified | 40 (0.4) |
| Upper GI | K92.0 | Hematemesis | 100 (1.1) |
|  | K92.1 | Melena | 260 (2.7) |
|  | I85.01 | Esophageal varices with bleeding | 40 (0.4) |
|  | I85.00 | Esophageal varices without bleeding | 10 (0.1) |
|  | K22.11 | Ulcer of esophagus with bleeding | 10 (0.1) |
|  | K25.0 | Acute gastric ulcer with hemorrhage | 10 (0.1) |
|  | K25.2 | Acute gastric ulcer with both hemorrhage and perforation | 0 (0) |
|  | K25.4 | Chronic or unspecified gastric ulcer with hemorrhage | 10 (0.1) |
|  | K25.6 | Chronic or unspecified gastric ulcer with both hemorrhage and perforation | 0 (0) |
|  | K26.0 | Acute duodenal ulcer with hemorrhage | 10 (0.1) |
|  | K26.2 | Acute duodenal ulcer with both hemorrhage and perforation | 0 (0) |
|  | K26.4 | Chronic or unspecified duodenal ulcer with hemorrhage | 10 (0.1) |
|  | K26.6 | Chronic or unspecified duodenal ulcer with both hemorrhage and perforation | 0 (0) |
|  | K27.0 | Acute peptic ulcer, site unspecified, with hemorrhage | 0 (0) |
|  | K27.2 | Acute peptic ulcer, site unspecified, with both hemorrhage and perforation | 0 (0) |
|  | K27.4 | Chronic or unspecified peptic ulcer, site unspecified, with hemorrhage | 0 (0) |
|  | K27.6 | Chronic or unspecified peptic ulcer, site unspecified, with both hemorrhage and perforation | 0 (0) |
|  | K28.0 | Acute gastrojejunal ulcer with hemorrhage | 0 (0) |
|  | K28.2 | Acute gastrojejunal ulcer with both hemorrhage and perforation | 0 (0) |
|  | K28.4 | Chronic or unspecified gastrojejunal ulcer with hemorrhage | 0 (0) |
|  | K28.6 | Chronic or unspecified gastrojejunal ulcer with both hemorrhage and perforation | 0 (0) |
|  | K29.01 | Acute gastritis with bleeding | 10 (0.1) |
|  | K31.811 | Angiodysplasia of stomach and duodenum with bleeding | 10 (0.1) |
|  | K31.82 | Dieulafoy lesion (hemorrhagic) of stomach and duodenum | 0 (0) |
| Lower GI | K55.21 | Angiodysplasia of colon with hemorrhage | 10 (0.1) |
|  | K62.5 | Hemorrhage of anus and rectum | 50 (0.5) |
|  | K92.2 | Gastrointestinal hemorrhage, unspecified | 110 (1.2) |
| Other | N02.0 | Recurrent and persistent hematuria with minor glomerular abnormality | 0 (0) |
|  | N02.1 | Recurrent and persistent hematuria with focal and segmental glomerular lesions | 10 (0.1) |
|  | N02.2 | Recurrent and persistent hematuria with diffuse membranous glomerulonephritis | 10 (0.1) |
|  | N02.3 | Recurrent and persistent hematuria with diffuse mesangial proliferative glomerulonephritis | 0 (0) |
|  | N02.4 | Recurrent and persistent hematuria with diffuse endocapillary proliferative glomerulonephritis | 0 (0) |
|  | N02.5 | Recurrent and persistent hematuria with diffuse mesangiocapillary glomerulonephritis | 0 (0) |
|  | N02.6 | Recurrent and persistent hematuria with dense deposit disease | 0 (0) |
|  | N02.7 | Recurrent and persistent hematuria with diffuse crescentic glomerulonephritis | 0 (0) |
|  | N02.8 | Recurrent and persistent hematuria with other morphologic changes | 10 (0.1) |
|  | N02.9 | Recurrent and persistent hematuria with unspecified morphologic changes | 10 (0.1) |
|  | K66.1 | Hemoperitoneum | 10 (0.1) |
|  | N93.8 | Other specified abnormal uterine and vaginal bleeding | 30 (0.3) |
|  | N93.9 | Abnormal uterine and vaginal bleeding, unspecified | 80 (0.8) |
|  | N95.0 | Postmenopausal bleeding | 0 (0) |
|  | R04.1 | Hemorrhage from throat | 10 (0.1) |
|  | R04.2 | Hemoptysis | 60 (0.6) |
|  | R04.8 | Hemorrhage from other sites in respiratory passages | 50 (0.5) |
|  | R04.9 | Hemorrhage from respiratory passages, unspecified | 20 (0.2) |
|  | R31 | Hematuria | 210 (2.2) |
|  | R58 | Hemorrhage, not elsewhere classified | 90 (1.0) |
|  | D68.3 | Hemorrhagic disorder due to circulating anticoagulants | 40 (0.4) |
|  | H35.6 | Retinal hemorrhage | 20 (0.2) |
|  | H43.1 | Vitreous hemorrhage | 10 (0.1) |
|  | M25.0 | Hemarthrosis | 10 (0.1) |
| FXa, factor Xa; GI, gastrointestinal.  ^a^Percentages calculated based on the total cohort (N = 9,470). | | | |
