# Supplementary material for: Anticoagulant prescribing trends, bleeding events, and reversal agent use in pediatric patients: A retrospective, real-world study
Source: PLoS One. 2025 May 8;20(5):e0323137. doi: 10.1371/journal.pone.0323137 (PMC12061172; doi:10.1371/journal.pone.0323137)
Supplement: S1 Fig — LMWH, low-molecular-weight heparin (includes danaparoid, tinzaparin, enoxaparin, dalteparin, and ardeparin); CDM, Clinformatics® Data Mart Database. Patient counts per anticoagulant therapy. Note that TriNetX rounds values up to the nearest 10 patients. (DOCX) [file pone.0323137.s007.docx]

**S1 Figure. Absolute number of pediatric patients stratified by anticoagulant prescription from 2007 to 2023 in TriNetX (A) and CDM (B)**


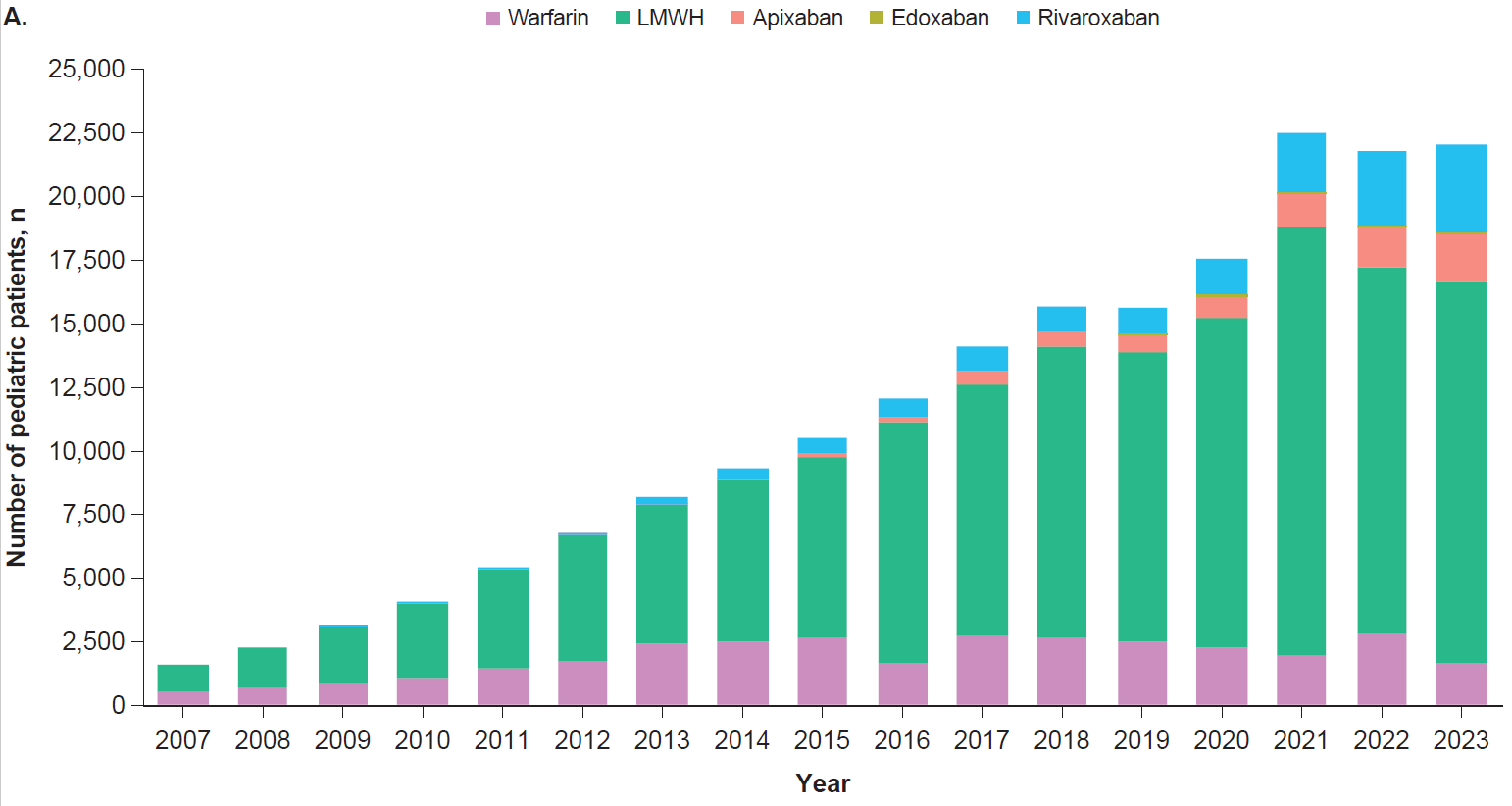


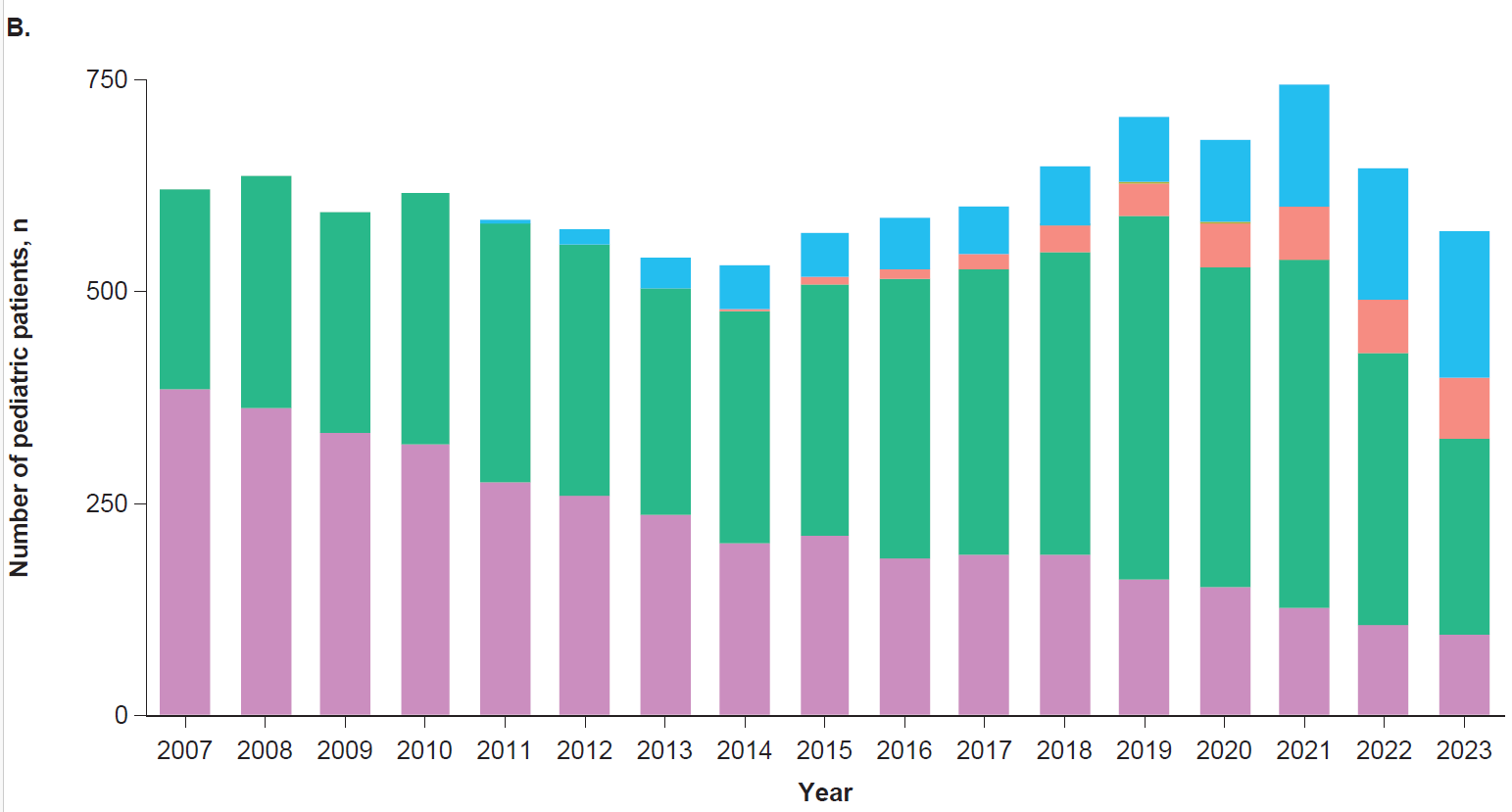


LMWH, low-molecular-weight heparin (includes danaparoid, tinzaparin, enoxaparin, dalteparin, and ardeparin); CDM, Clinformatics^®^ Data Mart Database.

Patient counts per anticoagulant therapy. Note that TriNetX rounds values up to the nearest 10 patients.
